# Substantial Reduction of Parenchymal Cerebral Blood Flow in Mice with Bilateral Common Carotid Artery Stenosis

**DOI:** 10.1038/srep32179

**Published:** 2016-08-18

**Authors:** Yorito Hattori, Jun-ichiro Enmi, Satoshi Iguchi, Satoshi Saito, Yumi Yamamoto, Kazuyuki Nagatsuka, Hidehiro Iida, Masafumi Ihara

**Affiliations:** 1 Department of Stroke and Cerebrovascular Diseases, National Cerebral and Cardiovascular Center, Suita, Osaka, Japan; 2 Department of Investigative Radiology, National Cerebral and Cardiovascular Center, Suita, Osaka, Japan; 3 Department of Regenerative Medicine, National Cerebral and Cardiovascular Center, Suita, Osaka, Japan

## Abstract

The bilateral common carotid artery stenosis (BCAS) mouse model, which replicates chronic cerebral hypoperfusion and white matter ischemic lesions, is considered to model some aspects of vascular cognitive impairment. Cerebral blood flow (CBF) changes in the brain surface post-BCAS have been demonstrated by laser speckle flowmetry, but CBF levels in the brain parenchyma remain unknown. Adult C57BL/6J male mice were subjected to BCAS using external microcoils. Brain magnetic resonance angiography (MRA) was conducted to visualize the intracranial main arteries while arterial spin labeling (ASL) was used to measure cortical and subcortical parenchymal CBF levels before and after BCAS. Brain MRA showed anterior circulation flow was substantially decreased until 14 days post-BCAS, which gradually but incompletely recovered over the following 14 days, with probable growth of collaterals from the posterior cerebral artery. ASL showed that cortical and subcortical parenchymal CBF remained decreased at approximately 50% of the baseline level during 1 and 14 days post-BCAS, recovering to approximately 70% at day 28. CBF levels in the parenchyma were lower than the cortical superficial region in the BCAS model and remained decreased without recovery during the first 2 weeks post-BCAS. These results suggest that the BCAS model reliably replicates chronic cerebral hypoperfusion.

Vascular cognitive impairment (VCI) is the second most common form of dementia after Alzheimer’s disease (AD)^1^, and the prevalence of mixed dementia with clinical and pathological features of both VCI and AD is increasing as a cause of age-related senile cognitive impairment^2^,^3^. Vascular factors are thought to be important mechanistic components of age-related cognitive impairment. To investigate the molecular mechanisms, several rodent models have been established^4^, including bilateral common carotid artery stenosis (BCAS) model^5^,^6^,^7^, reliably demonstrating chronic cerebral hypoperfusion, white matter ischemic damage (e.g., rarefaction and gliosis), and spatial working memory impairment. Using the BCAS model, previous reports have measured cerebral blood flow (CBF) in the brain surface containing subsurface microvessels in a depth of up to approximately 0.5 mm by laser speckle flowmetry (LSF) or laser doppler flowmetry^8^. Consequently, the CBF profiles in deep brain structures, such as the white matter and caudoputamen, after BCAS surgery remains unknown and, thus may not replicate those observed in subcortical VCI patients with low cerebral parenchymal CBF especially in the frontal lobe. We hypothesized that parenchymal CBF was lower than the brain surface or subsurface CBF after BCAS surgery and thus measured regional CBF levels using arterial spin labeling (ASL) magnetic resonance (MR) perfusion imaging and brain MR angiography (MRA).

## Results

### Physiological Parameters

Heart and respiratory rates were monitored during magnetic resonance imaging in all mice. Heart rates ranged 239‒578 beats per minute, and respiratory rates 30‒109 breaths per minute.

### Brain MRA

**Figure 1:**
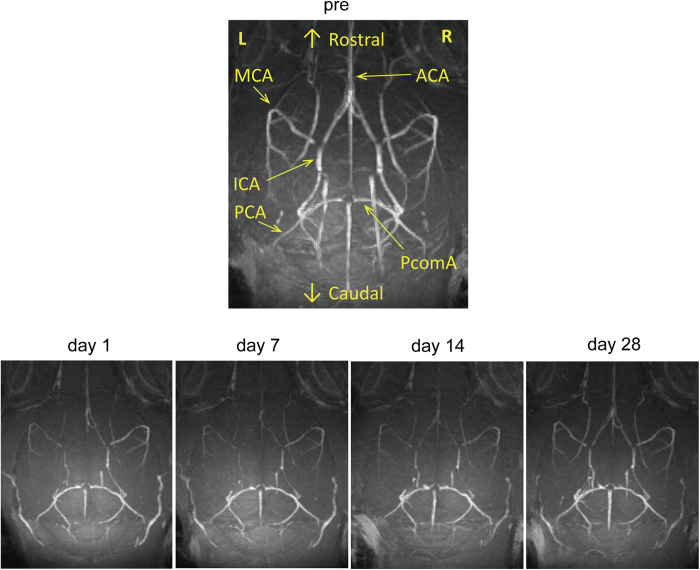
Intracranial arterial flow after bilateral common carotid artery stenosis (BCAS). Representative images of intracranial arterial flow obtained with a 7-tesla brain magnetic resonance angiography before and at 1, 7, 14, and 28 days after BCAS. Abbreviations: ICA: internal carotid artery; MCA: middle cerebral artery; ACA: anterior cerebral artery; PcomA: posterior communicating artery.

BCAS surgery showed that the MRA signal in the anterior circulation including the internal carotid artery, anterior cerebral artery and middle cerebral artery, substantially decreased from day 1 to 14 after surgery, with incomplete recovery of the signal over the following 2 weeks (Fig. 1). These results indicate that CBF in the anterior circulation was acutely reduced from day 1 after BCAS, but that cortical superficial CBF was more likely gradually compensated for by collateral growth from the posterior to anterior circulation.

### Temporal Profiles of Regional CBF Recorded by ASL

**Figure 2:**
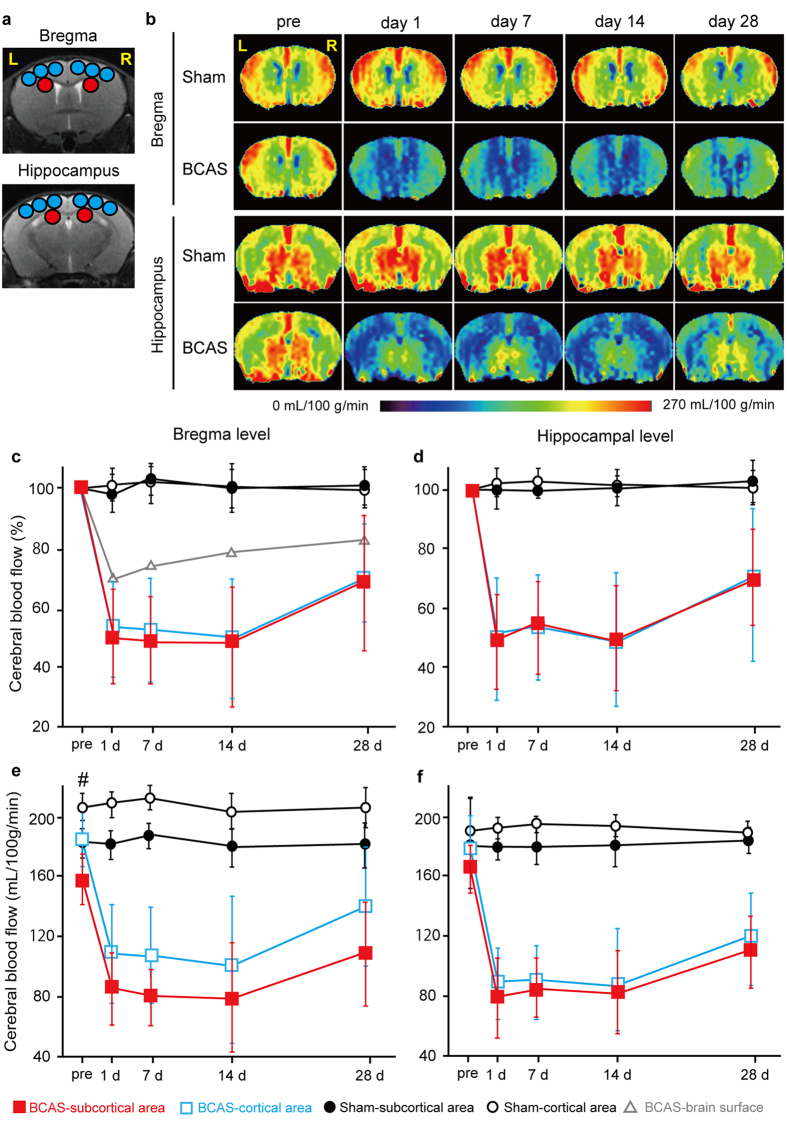
Temporal profiles of cerebral blood flow (CBF) of BCAS mice. (**a**) Regions of interest (ROIs) used in the analyses of cerebral blood flow (CBF) images obtained from arterial spin labeling (ASL) magnetic resonance perfusion imaging. The CBF values in cerebral cortex were calculated from the six blue ROIs, and red circles indicate ROIs of the subcortical area. (**b**) Representative multi-slice coronal CBF images obtained from ASL at the bregma and hippocampal levels. (**c**,**d**) Temporal profiles of CBF values which are presented as a percentage of the preoperative value in the cortical (open blue square) and subcortical (filled red square) parenchymal area of BCAS mice (n = 8), and in the cortical (black open circle) and subcortical (black filled circle) parenchymal area of sham-surgery mice (n = 5) at the bregma (**c**) and hippocampal level (**d**). For comparison, brain surface CBF profile (open gray triangle) at the bregma level before and after BCAS, assessed with laser speckle flowmetry in a previous report^9^, is superimposed (**c**). (**e**,**f**) Temporal profiles of CBF presented using absolute values (mL/100 g/min) in the cortical (open blue square) and subcortical (filled red square) parenchymal area of BCAS mice (n = 8) and in the cortical (black open circle) and subcortical (black filled circle) parenchymal area of sham-surgery mice (n = 5) at the bregma (**e**) and hippocampal level (**f**). Two-way repeated measures ANOVA indicates that there are significant differences in CBF values between BCAS vs. sham-surgery group in the cortical or subcortical area (p < 0.01) at the bregma and hippocampal levels (**c**‒**f**), and between cortical vs. subcortical area at the bregma level (p < 0.05; **e**). Unpaired t test indicates that there are significant differences between subcortical vs. cortical area pre-BCAS (^#^p < 0.05; **e**).

To explore CBF in the deep brain structures after BCAS, we performed ASL before and after BCAS (Fig. 2). Cortical and subcortical parenchymal CBF levels changed in a similar manner over 28 days after BCAS. We first evaluated temporal CBF profile in percentage with baseline set at 100%. At day 1, cortical and subcortical parenchymal CBF acutely decreased to 55.4% and 52.6% of their respective baseline levels in the coronal section at the bregma, and to 51.2% and 48.6% at the hippocampal level, remaining nearly unchanged until day 14. At 14 days, the cortical and subcortical parenchymal CBF levels were 52.1% and 52.0% of the respective baseline levels at the bregma, and 50.9% and 51.2% at the hippocampal level. At 28 days, the cortical and subcortical parenchymal CBF levels recovered to 71.1% and 69.8% of the respective baseline levels at the bregma, and to 70.3% and 69.9% at the hippocampal level; however, not indicating CBF significantly increased at day 28 compared to day 14 (day 14 vs. day 28; p > 0.05). The CBF was relatively unchanged over 28 days after sham surgery (Fig. 2c,d). Cortical surface CBF, assessed with LSF, showed reduction after BCAS surgery, but to a lesser degree in comparison to parenchymal CBF, and gradually recovered as early as day 1 (Fig. 2c)^9^. We subsequently evaluated absolute CBF values before and after BCAS or sham surgery. Subcortical CBF was significantly lower than cortical CBF before and after BCAS at the bregma (Fig. 2e) but not at the hippocampal level (Fig. 2f). Sham surgery induced only minimal changes of CBF, both at bregma and hippocampal levels (Fig. 2e,f).

## Discussion

In animal studies, CBF levels have been monitored using a variety of modalities including LSF, ASL, single photon emission computed tomography (SPECT), and ^15^O-positron emission tomography (PET). Among them, ASL may exert a discriminative role in assessing CBF noninvasively in animal studies including ours^10^,^11^,^12^, as the current ASL method is robust and ready to provide useful hemodynamic information^13^,^14^. In the present study, using ASL, we showed that the BCAS model maintained a significantly lower CBF for at least 14 days, especially in the subcortical area, faithfully modeling ‘chronic’ cerebral hypoperfusion. This was in contrast with a previous finding of cortical surface CBF using LSF, which showed gradual and continuous CBF recovery over 28 days after the operation (Fig. 2c)^9^. The quicker recovery of the cortical superficial CBF could result from leptomeningeal anastomosis from the posterior to the anterior circulation.

ASL, however, has some limitations; for example, it can suffer from the existence of prolonged transit time, i.e. the time it takes for tagged water to travel from the labeling position to the imaging slice, leading to an erroneous overestimation of CBF reduction^15^. This error is known to be particularly significant in humans because of the large size of the brain. By contrast, the small size of the mouse brain makes the transit time much smaller and the travel distance of tagged water much shorter than those in humans, minimizing the error^16^. Some studies have nevertheless reported an overestimation of CBF reduction even in rodent models of ischemia when conducted using 2.35 Tesla MRI^17^. As the present study was carried out at higher magnetic field strength of 7 Tesla, T_1_ relaxation time increases, resulting in less signal decay of tagged water during the traveling and relatively accurate estimation of CBF^15^. Therefore, the severity of hypoperfusion might have been somewhat overestimated but this could be minimal in the present study with no doubt about chronic cerebral hypoperfusion in the BCAS model.

The quick recovery of CBF assessed with LSF has been thought to be one of the drawbacks of the BCAS model because the temporal CBF profile is apparently different from that of VCI patients who suffer from continuous CBF reduction which can cause ischemic/hypoperfusive changes. However, the results of the present study indicate that cortical and subcortical parenchymal CBF levels are continuously reduced at least for 14 days, with lower levels in the subcortical area. Such continuous hypoperfusion moderate in severity at least for 14 days, may explain why white matter damage starts to develop at 14 days after the BCAS operation^5^. This model can therefore be used to explore potential VCI treatments that may increase CBF and restore cognitive impairment after chronic cerebral hypoperfusion.

## Methods

### Animals

Male C57BL/6J mice aged 10 weeks (weighing 22‒27 g; Japan SLC, Hamamatsu, Japan) were used and given access to food and water *ad libitum*. All animal experimental protocols were approved by the Institutional Animal Care and Use Committee at the National Cerebral and Cardiovascular Center, and were performed in accordance with the Guidelines for Proper Conduct of Animal Experiments established by Science Council of Japan. All procedures were performed under anesthesia and all efforts were made to minimize suffering.

### Study Design

C57BL/6J male mice were subjected to BCAS (n = 8) and sham surgery (n = 5). Temporal changes of CBF were measured with ASL (7 Tesla, BioSpec 70/30 USR; Bruker BioSpin, Ettlingen, Germany) and with brain MRA (BioSpec 70/30 USR) before and at 1 day, 7 days, 14 days, and 28 days after BCAS or sham surgery.

### Surgical Procedure of BCAS Surgery

Through a midline cervical incision, both common carotid arteries were exposed. Microcoils with an internal diameter of 0.18 mm (Sawane Spring, Hamamatsu, Japan) were applied to the bilateral common carotid arteries. Anesthesia was induced with 2% isoflurane and maintained with 1.5% isoflurane in 80% nitrous oxide and 20% oxygen. Rectal temperature was maintained between 36.5 °C and 37.5 °C (Supplementary Video 1).

### MRA and ASL

All MR scans such as MRA and ASL were performed using a 7-Tesla horizontal bore imaging system equipped with a gradient system capable of a maximum gradient amplitude of 669 mT/m and a slew rate of 7989 T/m/s as previously described with some modification^10^. The temporal change of subcortical CBF levels can be successfully monitored in a mouse hypoperfusion model after carotid surgery using ASL^10^. Radiofrequency transmission was performed using an 86-mm inner diameter volume coil. Signal was detected using a four-channel receive-only phased-array surface coil. The mice were anesthetized using isoflurane (4% for induction and 1.5–1.8% for maintenance) in 1.2 L/min room air mixed with 0.1 L/min oxygen. The animal was placed in a prone position, and the head fixed with a bite bar and ear bars. Body temperature was monitored by rectal thermometer and maintained with a warm waterbed and warm air. Heart rate and respiratory rate were continuously monitored.

Three-dimensional (3D) time-of-flight (TOF) MRA images were acquired using a fast low angle shot sequence with the following parameters: repetition time (TR)/echo time (TE), 22.43/3.30 ms; number of averages, 1; matrix size, 200 × 200 × 200; field of view (FOV), 2.0 × 2.0 × 2.0 cm^3^; spatial resolution, 100 × 100 × 100 μm^3^; and scan time, 11 min 13 s. In 3D TOF MRA, tilted optimized non-saturating excitation pulse and flow compensation were employed. Maximum intensity projection images were reconstructed using AZEWIN (AZE, Ltd., Tokyo).

CBF measurement of coronal slices was carried out using a flow-sensitive alternating inversion recovery technique^18^,^19^, an ASL-based method. In each of the non-selective and slice-selective experiments, twenty-two images with different inversion times were acquired using rapid acquisition with relaxation enhancement (RARE) sequence with the following parameters: RARE factor, 72; TR/TE, 10000/46 ms; number of averages, 1; matrix size, 128 × 128; FOV, 4.0 × 4.0 cm^2^; in-plane spatial resolution, 313 × 313 μm^2^; slice thickness, 1.0 mm; and number of slices, 1. The following inversion time values were used: 30, 100, 200, 300, 400, 500, 600, 700, 800, 900, 1000, 1100, 1200, 1300, 1400, 1500, 1600, 1700, 1800, 1950, 2100, and 2300 msec. Total scan time was 8 min 24 s. The CBF image was calculated from the obtained 44 images using ParaVison 5.1 (Bruker BioSpin). Absolute CBF values were calculated from T1 relaxation time difference between nonselective and slice-selective experiments. The CBF images of two coronal slices (bregma level and hippocampus level (bregma level −2.0 mm)) were acquired. Region of interest (ROI) analyses of CBF images were carried out using the Dr. View/LINUX R2.5.0 program (Asahi Kasei Information System, Tokyo). The ASL images were co-registered to the T2-weighted images for selection of ROIs by using the Dr. View/LINUX. In the corresponding slices of the *T*_*2*_-weighted image, circular ROIs with a diameter of 1 mm were symmetrically placed on cerebral cortex region and subcortical region including corpus callosum, caudoputamen and hippocampus, and superimposed on CBF images. CBF values were expressed as a percentage of pre-operative value.

As a reference, T2-weighted images were acquired using the RARE sequence with the following parameters: RARE factor, 8; TR/TE, 3500/28.74 ms; number of averages, 2; matrix size, 200 × 200; FOV, 2.0 × 2.0 cm^2^; in-plane spatial resolution, 100 × 100 μm^2^; slice thickness, 0.5 mm; gapless; number of slices, 35; and scan time, 2 min 55 s.

### Statistical Analysis

Statistical analysis was conducted using StatView (SAS Institute, Cary, NC, USA). All values are expressed as means ± standard deviation of the mean in the figures. Data were analyzed by unpaired t test or 2-way repeated measures ANOVA. Differences with *p* < 0.05 were considered statistically significant in all analyses.

## Additional Information

**How to cite this article**: Hattori, Y. *et al.* Substantial Reduction of Parenchymal Cerebral Blood Flow in Mice with Bilateral Common Carotid Artery Stenosis. *Sci. Rep.* **6**, 32179; doi: 10.1038/srep32179 (2016).

## Supplementary Material

Supplementary Information

Supplementary Video 1
