# Supplementary material for: Substantial Reduction of Parenchymal Cerebral Blood Flow in Mice with Bilateral Common Carotid Artery Stenosis
Source: Sci Rep. 2016 Aug 18;6:32179. doi: 10.1038/srep32179 (PMC4989493; doi:10.1038/srep32179)
Supplement: Supplementary Information [file srep32179-s1.pdf]

Supplementary information

Substantial reduction of parenchymal cerebral blood flow in mice with bilateral common carotid artery stenosis

Yorito Hattori, Jun-ichiro Enmi, Satoshi Iguchi, Yumi Yamamoto, Satoshi Saito, Kazuyuki Nagatsuka, Hidehiro Iida, Masafumi Ihara

Corresponding author: Masafumi Ihara; Telephone, (+81)-6-68335012; Fax, (+81)-6-68355137; E-mail, ihara@ncvc.go.jp

Supplementary video legend

Supplementary Video 1: Surgical implantation of a microcoil

Through a midline cervical incision, a left common carotid artery (CCA) is exposed and freed from their sheaths including left vagus nerve. A 4-0 silk suture is placed around the CCA. The artery was gently lifted by this suture, the microcoil is applied surgically to the CCA, and the suture is removed.
